# miR-154 targeting ZEB2 in hepatocellular carcinoma functions as a potential tumor suppressor

**DOI:** 10.3892/or.2021.8113

**Published:** 2021-06-14

**Authors:** Xiaoli Pang, Kexin Huang, Qianqian Zhang, Yujiao Zhang, Junqi Niu

Oncol Rep 34: 3272-3279, 2015; DOI: 10.3892/or.2015.4321

Following the publication of this paper, it was drawn to the Editors’ attention by a concerned reader that certain of the western blotting data shown in Fig. 3C, 4D, 5A and 6D bore unexpected similarities to data appearing in different form in other articles by different authors. Owing to the fact that the contentious data in the above article had already been published elsewhere, or were already under consideration for publication, prior to its submission to *Oncology Reports*, the Editor has decided that this paper should be retracted from the Journal. After having been in contact with the authors, they agreed with the decision to retract the paper. The Editor apologizes to the readership for any inconvenience caused.

